# Exploring the factors influencing meaningful engagement of persons living with advanced dementia through the Namaste Care Program: a qualitative descriptive study

**DOI:** 10.1177/26323524231165319

**Published:** 2023-04-01

**Authors:** Marie-Lee Yous, Sheila A. Boamah, Paulette V. Hunter, Esther Coker, Thomas Hadjistavropoulos, Tamara Sussman, Sharon Kaasalainen

**Affiliations:** School of Nursing, Faculty of Health Sciences, McMaster University, 1280 Main Street West, Hamilton, ON L8S 4K1, Canada; School of Nursing, Faculty of Health Sciences, McMaster University, Hamilton, ON, Canada; Department of Psychology, St. Thomas More College, University of Saskatchewan, Saskatoon, SK, Canada; School of Nursing, Faculty of Health Sciences, McMaster University, Hamilton, ON, Canada; St. Peter’s Hospital, Hamilton Health Sciences, Hamilton, ON, Canada; Department of Psychology and Centre on Aging and Health, University of Regina, Regina, SK, Canada; School of Social Work, McGill University, Montreal, QC, Canada; School of Nursing, Faculty of Health Sciences, McMaster University, Hamilton, ON, Canada

**Keywords:** dementia, engagement, long-term care, Namaste Care, psychosocial intervention

## Abstract

**Background:**

Meaningful engagement has been described as active participation based on a person’s interests, preferences, personhood, or perceived value. It has many benefits for persons living with dementia in long-term care (LTC) homes, including improvement in physical and cognitive function, and mental health. People with advanced dementia continue to need and benefit from inclusion and social contact in LTC, yet there is not a well-developed understanding of how to support this. A tailored intervention called Namaste Care has been shown to be an effective approach to meaningfully engage residents in LTC, decrease behavioral symptoms, and improve their comfort and quality of life. There is a need to consider how best to deliver this intervention.

**Objective:**

The aim of this study was to describe environmental, social, and sensory factors influencing meaningful engagement of persons with advanced dementia during Namaste Care implementation in LTC.

**Methods:**

In this qualitative descriptive study, focus groups and interviews were conducted with families, volunteers, staff, and managers at two LTC homes. Directed content analysis was conducted. The Comprehensive Process Model of Engagement was used as a coding framework.

**Results:**

With respect to environmental attributes, participants emphasized that a designated quiet space and a small group format were helpful for engagement. In terms of social attributes, participants emphasized Namaste Care staff capacity to deliver individualized care. Regarding sensorial factors, familiarity with the activities delivered in the program was emphasized.

**Conclusion:**

Findings reveal the need to offer small group programs that include adapted recreational and stimulating activities, such as Namaste Care, for residents at the end of life in LTC. Such programs facilitate meaningful engagement for persons with dementia as they focus on individual preferences, comfort, and inclusion while recognizing changing needs and abilities of residents.

## Introduction

More than 55 million people are living with dementia worldwide with projected increase of 10 million new diagnoses per year.^1^ According to the 2021 Canadian Census, of the nearly 200,000 individuals living in long-term care (LTC) homes,^2^ 69% have a diagnosis of dementia.^3^ Persons with dementia in LTC homes require consistent intellectual stimulation and social interaction with family, friends, and the wider community to improve and sustain their quality of life.^4^ Positive stimulation and social connections are key in addressing behavioral expressions, such as agitation and social withdrawal,^5^ and slowing the progression of dementia.^6^ Prolonged lack of stimulation for persons with dementia in LTC homes can lead to apathy, depression, boredom, and isolation.^7,8^ Evidence from observational and longitudinal studies has shown low social participation to be associated with greater loneliness, and an increased risk of acquiring dementia.^6,9^

To support quality of life, persons with dementia require opportunities for meaningful engagement that consider their needs, preferences, and abilities and are tailored to their specific stage of dementia.^10^ Residents with dementia in LTC require comprehensive care that extends beyond physical needs, such as those related to hygiene care and nutritional provision. In particular, people with moderate to advanced dementia require cognitive stimulation and meaningful engagement in activities as they are at increased risk for social isolation and withdrawal from activities due to pronounced reduction in their ability to socialize with others.^11–13^ Meaningful engagement has been found to be a potential protective factor against isolation and cognitive decline.^14^

People with advanced dementia continue to need and benefit from inclusion and social contact, yet there is not a well-developed understanding of how to support this. A new tailored intervention, named Namaste Care, has been shown to be an effective approach to meaningfully engage residents in LTC, decrease behavioral symptoms, and improve their comfort and quality of life.^15–17^ Despite its potential, few studies have explored the benefits and principles of Namaste Care with residents and staff in LTC.

### Meaningful engagement

Meaningful engagement has commonly been described as active participation based on a person’s interests, preferences, personhood, or perceived value.^18–21^ Engagement in meaningful activities has many benefits for LTC residents, including improvement in physical and cognitive function, and mental health.^22^ Meaningful engagement can improve anxiety, depression, and behavioral expressions, which are often experienced by residents with dementia in LTC homes.^20,23^ Despite its importance, meaningful engagement is lacking for persons with dementia in LTC homes.^24^

As persons with dementia progress toward end-stage dementia, they become increasingly reliant on their physical and social environment to support their engagement.^25^ As such, it is important to better understand how staff in LTC homes view meaningful engagement for positive well-being among persons with dementia. Meaningful engagement requires a comprehensive assessment of the environment in which programs are delivered and the response of persons with advanced dementia to stimuli. Without a rich understanding of how programs work and in which context, there is a risk of continuing to provide passive activities that can lead to further decline in health and well-being of residents. In this study, we applied the Comprehensive Model of Engagement Framework^8^ to contribute to our understanding of how the Namaste Care program can promote meaningful engagement for people with advanced dementia living in two LTC homes.

### Comprehensive model of engagement framework

**Figure 1.**
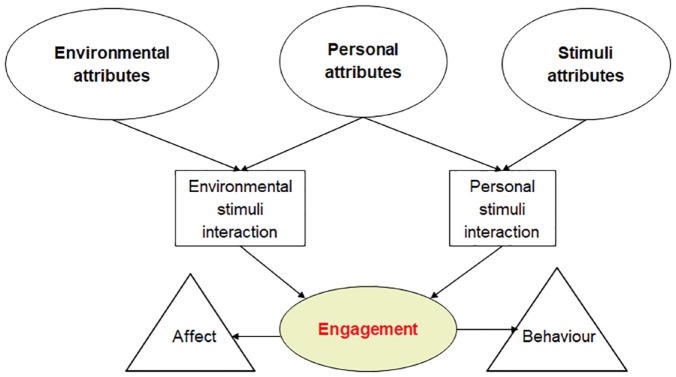
Adapted version based on the comprehensive model of engagement framework (Cohen-Mansfield *et al.*^8^).

The Comprehensive Model of Engagement framework provides a rigorous structure for assessing meaningful engagement of persons with dementia.^8^ Within this framework, engagement with a stimulus is influenced by (1) environmental, (2) person, and (3) stimulus attributes (see Figure 1 for an adapted version). *Environmental attributes* consist of contextual elements, such as the location of stimulus presentation, the number of individuals present (e.g. staff, other residents, and family care partners), noise, temperature, and light levels, time of day, and the approach used to introduce a stimulus (e.g. gradually with modeling or abruptly). *Person attributes* are individual characteristics of persons with dementia which may impact their engagement with a stimulus. These include cognitive abilities, and past interests and life stories. *Stimulus attributes* are most likely to positively affect the level of engagement based on the presence of social qualities and degree of manipulation that allows an individual to arrange or explore an object through touch. These attributes also interact with one another. For example, certain stimuli can be easily affected by the setting in which it is being delivered, which is important to consider when implementing Namaste Care. If a group program is delivered in a space with excessive noise and distractions, this can impact the level of engagement of persons with dementia. Person-tailored stimuli are more likely to lead to higher levels of engagement because they reflect individual stories, abilities, and preferences. Delivering more than one type of stimulus in group settings with time built in for one-on-one interactions has been found to facilitate engagement.^26^ When engagement is achieved, this can lead to a change in affect among persons with dementia that determines how behavioral expressions are presented.^8^ Given that environmental, person, and stimulus attributes affect meaningful engagement of residents with dementia, it is important to explore these concepts within the delivery of a well-established program, such as Namaste Care.

### Namaste Care

Namaste Care is a multisensory, person-centered program that advocates a slow pace, high-touch approach, and sensorial activities designed for advanced dementia.^17^ It was originally created in 2003 for use in LTC to improve the quality of life of persons with advanced dementia. Namaste Care is a complex intervention consisting of psychological, social, and spiritual components that can be flexibly applied to support LTC residents with different needs and preferences.^27^ The program is delivered in a comfortable environment using an unhurried, loving-touch approach.^17^ Various meaningful engagement activities are provided within the Namaste Care program, including music therapy, massages, socialization, aromatherapy, and snacks.

In LTC homes, Namaste Care sessions are delivered by trained care providers in a private room with soft lighting and no interruptions. Prior to the session, a Namaste Care box or cart is created which contains tailored items, such as lotions, life-like dolls, plush animals, photos, and sensory balls. The sessions are delivered 2 h at a time in the morning and afternoon, and encourage active participation of able family members. As part of the Namaste Care program, personalized music is played and scents, such as lavender, are diffused. Activities are gradually introduced by care providers, one at a time, and include touch that is used to connect staff with persons with dementia through hand/foot massages, applying lotions, and hair brushing. Nutrition is an important component of the program and persons with dementia are provided with food and drink based on their preferences and swallowing abilities. Persons with dementia are monitored by healthcare providers for signs of pain and discomfort during the sessions.^17^ With regard to training LTC staff responsible for delivering Namaste Care in this study, staff received written resources and a 2-h in-person training session to familiarize them with the equipment (e.g. projector, computer tablet), review processes, and answer questions. Each staff member received a copy of Simard’s book, ‘The End-of-Life Namaste Care Program for People with Dementia’.^17^ Each Namaste Care session was facilitated by a single staff member (e.g. personal support worker, nurse, or activity aide) with the support of one volunteer.

Namaste Care has been used worldwide and in different settings, including LTC, hospice, acute care, and home settings.^17,28^ Positive findings of the program include reduced use of anti-anxiety and psychotropic medications, lower risk of delirium, decreased pain, and improvement in quality of life for persons with dementia and relationships with staff.^23,29–32^ Family members reported being more relaxed during interactions with persons with dementia following Namaste Care.^23^

The importance of meaningful engagement of persons with advanced dementia in LTC homes is well-established.^8,20^ Despite the critical need to engage persons with advanced dementia, few studies have considered the context of program delivery which has a significant impact on the level of engagement of persons with dementia. To date, no study has explored the Namaste program within an engagement framework to determine how best to deliver it in LTC homes and how it can promote meaningful engagement. Our goal was to address this gap by providing a comprehensive description of environmental, social, and sensory factors that influence meaningful engagement of persons with advanced dementia in the Namaste Care program in LTC homes guided by the Comprehensive Model of Engagement Framework.^8^

## Methods

### Study design

This study used a qualitative descriptive design^33^ guided by directed content analysis,^34^ which was chosen to reach a fundamental understanding of how Namaste Care can be applied in LTC to promote engagement of residents using language that reflects everyday experiences of participants.^33,35^ Qualitative description considers language as a way of communicating straight descriptions with some room for interpretation.^33,35^ The COnsolidated criteria for REporting Qualitative research (COREQ) checklist was used as a reporting guideline.^36^ This study was part of a larger mixed-methods study evaluating the feasibility, acceptability, and effects of the Namaste Care program on resident outcomes (i.e. quality of life, pain, and neuropsychiatric symptoms) and family care partner outcomes (i.e. role stress and quality of visits).

### Setting

Two non-profit LTC homes located in urban areas of Southern Ontario, Canada were selected. The characteristics of the homes differ with regard to the number of beds available (e.g. fewer than or greater than 200 beds) and type of services offered (e.g. supportive housing, dementia care). One home was a large residential LTC home (just under 300 beds) and the other home was a medium-sized LTC home (120 beds). Both of these LTC homes had a high proportion of residents with moderate to advanced dementia. There was strong leadership interest in implementing Namaste Care in the LTC homes selected as evidenced by administrators providing necessary in-kind resources.

### Recruitment and sample

LTC administrators, staff including nurses, personal support workers/nursing aides, housekeepers, recreation programmer, nutrition manager, volunteers, and family members of residents were invited by the research team by phone or email to participate in interviews or focus groups. The inclusion criteria were (a) 18 years or older; (b) able to speak, read, and understand English; and (c) affiliated with site 1 or 2 as a staff, volunteer, or family member of a resident aged 65 years and older with moderate to advanced dementia. Purposeful sampling, specifically maximum variation sampling,^37^ was used to target diverse participants with regard to roles and responsibilities in the planning and implementation of Namaste Care. For example, LTC administrators were involved in scheduling sessions while frontline staff, such as personal support workers and nurses, were responsible for facilitating sessions. Staff from various disciplines and levels of experience working in a LTC home were sought. The perspectives of family members of residents were also sought to clarify their interpretation and perceived value of the Namaste Care program.

### Data collection

Data were collected from October 2017 to April 2018 at each LTC home within approximately the first 6 months of implementation of Namaste Care. Focus groups and interviews were conducted by phone or in person at the LTC home by six trained research assistants. These lasted from 30 to 60 min. A total of 8 focus groups and 22 individual interviews were completed. Focus groups with four to nine staff members per group were held. The total number of participants for both sites was 68. The research team organized focus groups based on similar disciplines. For example, there were focus groups specifically for nurses, personal support workers, and volunteers. For those not fitting into the three categories, focus groups were held with other service providers, such as recreation programmers, nutrition managers, and housekeepers. Individual interviews with family members, volunteer coordinators, and directors of care were conducted due to scheduling conflicts and preferences of participants. A token of appreciation in the form of a CAD$15 gift card was provided to everyone who participated in the study. Questions about how the Namaste Care program engages, families, residents, and staff in relation to environmental, social, and sensory factors were asked. All focus groups and interviews were audio-recorded with permission, transcribed by a trained transcriptionist, and reviewed for accuracy by graduate students. Field notes were made during and after the interviews. Data collection continued until data saturation was reached and no new themes emerged. The focus group and interview guides are available on request.

### Data analysis

Consistent with qualitative description,^33^ directed content analysis was used to guide the development of initial codes by starting with the Comprehensive Model of Engagement framework^8^ as an analytic lens.^34^ Deductive coding was used to develop themes based on a coding tree created from the Comprehensive Model of Engagement framework.^8^ Constant comparative analysis was used to identify commonalities and differences within the focus group and interview data. Person and stimulus attributes were renamed to social and sensory attributes to better fit the data after a read of all transcripts prior to initial coding. It is recommended to make adjustment to pre-existing frameworks to create greater alignment between data and categories.^33^ Two members of the research team (S.A.B. and M-L.Y.) reviewed transcripts. M-L.Y. conducted coding independently with regular meetings with S.A.B. to review the development of codes and resulting themes. Themes were reviewed by all members of the research team for consensus. Data analysis was conducted using NVivo data management software.^38^

### Rigor and trustworthiness

To uphold rigor and trustworthiness in qualitative research, strategies were implemented to address Lincoln and Guba’s trustworthiness criteria: credibility, transferability, dependability, and confirmability.^39^ Investigator triangulation was used to ensure credibility of findings. Feedback from all members of the research team was sought as they hold expertise in LTC, palliative care, and dementia care research. This strategy was also found to complement and support the validation of data.^39^ Comprehensive and detailed descriptions of the setting and sample of the study were provided to uphold transferability of findings.^39^ The research team ensured that processes of the study were informed logical by conducting a comprehensive review of the existing literature to determine gaps in the literature.

## Results

### Demographics

There were 31 participants from site 1 and 37 from site 2. LTC staff and administrators at both sites varied in ages. Volunteers at site 2 were all 65 years and older while at site 1, volunteers varied in ages. At both sites, more than half of the participants were female – 74.2% at site 1 and 70% at site 2. See Table 1 for demographic characteristics of participants at respective sites.

**Table 1.** Demographic characteristics for site 1 (*n* = 31) and site 2 (*n* = 37).

Category	Site 1	Site 2
	*n* (%)	*n* (%)
Age (years)		
Nurses	*n* = 5	*n* = 11
25–34	2 (40)	4 (36.4)
35–44	1 (20)	1 (10)
45–54	1 (20)	2 (18.2)
55–64	1 (20)	1 (10)
No response	N/A	3 (27.3)
Personal support workers	*n* = 5	*n* = 6
35–44	1 (20)	3 (50)
45–54	3 (60)	1 (16.7)
55–64	1 (20)	2 (33.3)
Other service providers (e.g. recreation programmers, nutrition managers, and housekeepers)	*n* = 5	*n* = 4
<25	N/A	2 (50)
25–34	1 (20)	1 (25)
35–44	1 (20)	1 (25)
55–64	3 (60)	N/A
Administrators	*n* = 7	*n* = 5
35–44	N/A	2 (40)
45–54	3 (42.9)	2 (40)
No response	4 (57.1)	1 (20)
Volunteers	*n* = 4	*n* = 6
<25	1 (25)	N/A
55–64	1 (25)	N/A
⩾65	2 (50)	6 (100)
Family members	*n* = 5	*n* = 5
45–54	1 (20)	N/A
55–64	2 (40)	3 (60)
>65	2 (40)	2 (40)
Sex		
Nurses	*n* = 5	*n* = 11
Female	5 (100)	6 (54.5)
Male	N/A	3 (27.3)
No response	N/A	2 (18.2)
Personal support workers	*n* = 5	*n* = 6
Female	4 (80)	5 (83.3)
Male	1 (20)	N/A
Other	N/A	1 (16.7)
Other service providers (e.g. recreation programmers, nutrition managers, and housekeepers)	*n* = 5	*n* = 4
Female	3 (60)	2 (50)
Male	2 (40)	1 (25)
No response	N/A	1 (25)
Administrators	*n* = 7	*n* = 5
Female	3 (42.9)	4 (80)
No response	4 (57.1)	1 (20)
Volunteers	*n* = 4	*n* = 6
Female	3 (75)	5 (83.3)
Male	1 (25)	1 (16.7)
Family members	*n* = 5	*n* = 5
Female	5 (100)	4 (80)
Male	N/A	1 (20)
No. of years working in site-specific LTC, *M* (*SD*)		
Nurses	11.6 (3.2)	7.6 (9.2)
Personal support workers	3.0 (6.5)	12.3 (11.5)
Other service providers (e.g. recreation programmers, nutrition managers, and housekeepers)	9.0 (5.7)	2.5 (1.7)
Administrators	6.7 (6.4)	7.8 (5.9)
No. of years volunteering in site-specific LTC, *M* (SD)		
Volunteers	3.4 (3.2)	Not available
No. of years volunteering in LTC, *M* (*SD*)		
Volunteers	2.3 (0.5)	3.3 (1.2)
No. of years loved ones of family members in LTC, *M* (*SD*)		
Residents	3.1 (4.0)	2.8 (1.6)

LTC, long-term care; N/A, not applicable.

The total counts do not add up to 100% due to rounding.

### Themes

In this section, the themes pertaining to environmental, social, and sensory attributes are discussed within the context of the Namaste Care program for residents with advanced dementia in LTC. There were very few differences between sites with regard to considerations for meaningful engagement of residents with advanced dementia.

#### Environmental attributes

Two themes were identified with respect to environmental attributes: (a) having a dedicated and quiet space for Namaste Care is important and (b) a small group setting enhances opportunities for companionship. Participants at both sites perceived that Namaste Care requires careful planning and consideration regarding where it should be delivered, so there is reduced potential for disruptions.

*Having a dedicated and quiet space for Namaste Care is important*. Staff perceived that residents required their own private space away from usual distractions within LTC homes. As such, staff felt that the Namaste Care program sessions should be held in a private and quiet room to create a calming environment:

I think it allows them a quieter space. I think sometimes the background noises, the carts being pushed, other people being disruptive can get to them, but I find when they’re in the Namaste Room they can connect a bit more, it’s a smaller space, it’s more relaxed, they’re not as agitated and they start to kind of look out for each other. (Site 2 – Community Center Staff)

Providing the Namaste Care program in a special environment was perceived by staff as enabling greater connections between residents as they become less agitated and less socially isolated. Namaste Care should not be delivered in common areas, such as the dining room, or next to noisy environments, such as the nursing station.

*A small group setting enhances opportunities for companionship*. Staff and family members perceived that residents with advanced dementia required individualized care within a small group setting. They were perceived as often being left out of traditional LTC recreational programs due to greater cognitive and physical challenges compared to other residents. Although Namaste Care is often delivered in a small group setting in LTC, the person delivering the program is expected to spend some time with each resident on a one-on-one basis to engage them in preferred activities that meet their individual needs. Simply being surrounded by others, including other residents and staff, created a sense of companionship and community:

So, we bring all the residents together and we encourage socialization whether it’s through music and everybody sitting there together. The staff will sit next to residents and hold their hands or just encourage. You know we encourage them together in a group setting. So definitely socialization is encouraged. (Site 2 – Volunteer Coordinator)

This creation of a community for residents with advanced dementia ensured that they were offered programs that support their quality of life and increase a sense of belonging.

#### Social attributes

The meaningful engagement of residents with advanced dementia in LTC through the Namaste Care program necessitated prior knowledge of their preferences, abilities, and needs. Themes developed under social attributes were (a) capacity of Namaste Care staff to individualize care was key for residents with advanced dementia and (b) families provide important information that can help tailor activities to residents’ needs and abilities.

*Capacity of Namaste Care staff to individualize care was key for residents with advanced dementia.* Staff and families perceived the need to ensure that those delivering Namaste Care know the residents well to effectively engage them. Families of residents stressed the importance of consistency in delivering the Namaste Care program so the same group of individuals are delivering the program every time. This ensures that they have adequate knowledge of residents and what works well for each resident during the Namaste Care sessions:

I like the part where you said about the same people and then they get to know their patient basically. That way they know what buttons they can push with them. What they do like, what they don’t like. So that is I think very important. Not to interchange people all the time. So that would work. I think it sounds like a nice idea about the room. (Site 1 – Family Member)

The success of the Namaste Care program was perceived as being dependent on the ability of Namaste Care staff to offer individualized interactions for residents. The program was perceived as being different than usual care as Namaste Care staff are interacting with residents on a personal level outside of care and mealtimes and using creative approaches to engage them:

Well this is I think where prior to the Namaste Program other than sort of wheeling them [residents in advanced stages of dementia] to things that they couldn’t possibly participate in we didn’t have that much and so the implementation of the Namaste has really helped to engage them in an active way. Which is mainly sort of a sensory stimulation environment. So that program for sure would be our main way of you know formalized group kind of way engaging those residents. (Site 2 – Administrator)

*Families Provide Important Information That Can Help Tailor Activities to Residents’ Needs and Abilities.* For residents with advanced dementia who would most likely not be able to effectively communicate their needs verbally, family members were perceived by staff as important sources of information related to residents’ histories, life stories, and preferences. Staff perceived a gap in their own knowledge of the residents as persons. Families therefore played a key role in providing information about the favorite activities of residents and bring in familiar objects to engage them:

Another big, important thing when you’re looking to plan programs for these folks is to not be afraid to you know utilize the family members. Get any information that you can about that person. Things like this person loved cats. Sometimes the littlest things can help. Any background information. Things that they absolutely hated. Maybe they loved working on cars so it could be as simple as bringing in a book with pictures of old cars. The family is really key in those regards because with these folks they’re not going to be able to tell you things that they love or hate. So just using the family members as much as you can to get any of that information. (Site 2 – Volunteer Coordinator)

Family members of residents were sometimes underused as sources of knowledge about residents yet were easily accessible to staff and often considered as partners in care. Staff perceived that no matter how small a piece of information may seem it still has the potential to be used to engage residents and inform the selection of activities to enhance their quality of life.

#### Sensory attributes

The sensory attributes that were perceived as most influential in leading to positive changes in residents with advanced dementia were found in activities that targeted more than one basic human sense (e.g. touch, smell, taste, hearing, sight) and provided a source of comfort. Themes were as follows: (a) activities that targeted multiple senses led to positive effects in residents with advanced dementia and (b) activities selected should provide comfort and distraction for residents.

*Activities that targeted multiple senses led to positive effects in residents with advanced dementia.* Staff, families, and volunteers perceived that Namaste Care ensures that residents are provided with more than one activity to increase their likelihood of engagement. Providing touch-based activities, such as massages, range of motion activities, and fidget blankets (e.g. blankets with items attached that vary in texture, color, and size to provide stimulation), was perceived by families and staff as creating a sense of physical connection with the social and physical environment for residents with advanced dementia. Range of motion activities was perceived as also leading to clinical benefits for residents, such as decreased rigidity:

I always think back to when [name] was here as well and one of our residents [name], he’s passed away now but how we got him to kind of help with his range of motion. So we got him to open up his hand all the way so I thought that was really amazing. That’s when we first started the Namaste Program but just even being able to do little things like that. . . ..if someone was in there and able to massage their hands and like help with their range of motion. The families could be shocked and happy in the sense of oh wow they can still move that part of their body or things like that. That’s pretty amazing. (Site 2 – Community Center Staff)

Music was perceived by staff and family members as a powerful tool in the Namaste Care program to lift the spirits of residents and engage them in familiar songs. Playing familiar songs for residents increased the likelihood of engagement by singing along to songs and greater attempts at verbal expression:

They would play music she loved. I had her in there actually, we just sat down together and they had music up on the screen, it was kind of like a sing along so I could actually see the words. And then she could hear the songs and I could see her moving her mouth. (Site 2 – Family Member)

Residents in the Namaste Care program were provided with soothing audiovisual stimulation, such as videos and movies. Staff would also engage residents in uplifting conversations and read books out loud for residents. This type of stimulation was perceived as decreasing agitation and responsive behaviors among residents. Residents with advanced dementia were provided with opportunities to leave their bland rooms and enter a space where all of their senses are being stimulated:

. . .the family feels that their person is participating in a program and that person is leaving their room for a change of scenery and there are some wonderful things in the Namaste Room. Which is also stimulating them. I don’t know if you’ve seen the room we have machines with coloured lights or it’s soft music or the TVs on with like specialized programs that they put on and you can see that the resident is actually. . .you know through expression or through participation you can see they look a bit more relaxed and also the staff and the volunteers who are in there can see the situation or see if the person is agitated or if they don’t want to be there and then they take them back home to their room. . .I think that for the most part that people enjoy it and the residents are benefiting from it. (Site 2 – Volunteer Coordinator)

*Activities selected should provide comfort and distractions for residents.* The types of activities provided for residents (e.g. aromatherapy, massages, applying lotion, music) were perceived by family members and staff as making residents feel comfortable and distracted from feelings of pain or boredom. ‘I think that it could possibly ease people’s pain. Either from the massage itself or even just as a distraction. Sometimes you can’t take away all of the pain. So, distraction techniques and using other non-pharmacological things is beneficial’ (Site 1 – Resident Care Supervisor). Namaste Care was perceived as an effective non-pharmacological approach to support the quality of life of persons with advanced dementia. Namaste Care promoted a sense of inclusion for residents and let them know that there are others who care for their well-being:

I was really pleased that the Namaste program came along because I was feeling like it was to sort of put her [mother] aside because there were others that were maybe more needy or maybe more responsive and a few times I’ve said to the Recreation Staff for example, you know even though mom can’t sing anymore or she can’t clap her hands she still likes to be involved in you know sort of group activities. She still really appreciates or really enjoys that. (Site 2 – Family Member)

## Discussion

This study is unique in its use of the Comprehensive Model of Engagement framework^8^ to explore the influence of interactions among environmental, social, and sensory factors on meaningful engagement of residents with advanced dementia participating in the Namaste Care program. Key findings to support the meaningful engagement of persons with advanced dementia include: (a) creating a private environment for Namaste Care which can promote social interactions; (b) ensuring that participants in the program receive tailored activities that resonates with their life stories and preferences; and (c) providing them with activities that target multiple senses with the ultimate purpose of creating comfort.

In this study, a dedicated and quiet space was found to promote meaningful engagement of residents with advanced dementia in Namaste Care. Careful consideration of environmental factors, such as noise levels and the designated area where sessions were to take place, was important for the success of the program.^17^ The findings suggest that it was not perceived as suitable to simply convert a part of the dining room to hold Namaste Care sessions or have the sessions near the busy nursing station out of convenience. In support of the study findings, physical contexts in LTC have been found to require adaption to compensate for the abilities of residents with dementia to meaningfully engage in activities.^4^ Consistent with other studies,^4,40^ our findings highlight the fact that there are multiple environmental barriers that can limit the participation of residents with dementia in meaningful activities which are often taken for granted, such as lighting, physical space, and sound. Environmental adaptations should reflect the cognitive function and tolerance of residents to stimuli by reducing distractions as much as possible.^4^ Environmental modifications have the potential to increase the length of time that residents can engage in programs. Staff in LTC have a role in assisting residents with advanced dementia to meaningfully engage in social activities that promote their quality of life. Diverse and multiple approaches to engage residents with advanced dementia take concerted effort by staff so residents are not disadvantaged with regard to meaningful engagement.^24^

In the current study, residents with advanced dementia in LTC benefited from Namaste Care sessions delivered by Namaste Care staff who were familiar with their life stories, preferences, and abilities. Participants reported the importance of providing individualized care for residents with advanced dementia through the Namaste Care program. Families filled an important gap in sharing information about residents with Namaste Care staff. Namaste was found to work best within a context wherein relational care is valued and supported. Du Toit *et al.*^4^ also found that staff familiarity with residents’ stories was particularly important as persons with advanced dementia may no longer be able to verbally communicate and families are the next best source of knowledge. Kemp *et al.*^24^ found similar strategies to promote meaningful engagement of persons with dementia in LTC homes, including knowing the individual, recognizing that the needs and abilities of persons with dementia change from day to day, and seeing all encounters as opportunities for interactions initiated by various individuals (e.g. family members, kitchen staff, and housekeeping). Tailored or person-centered activities reflecting functional abilities, life stories, and personalities promote meaningful engagement among persons with advanced dementia.^28,40,41^ Study findings have implications for ensuring that volunteers supporting staff in delivering Namaste Care are also aware of the stories and preferences of residents.

A unique contribution of this study is that findings reveal that it is possible to provide personalized care for residents with advanced dementia within a small group setting. Findings of the current study provide support for Namaste Care, a small group program, which can effectively use existing LTC resources (e.g. staffing, equipment, space) to provide meaningful engagement to a greater number of residents with advanced dementia in one session. Others have found that group programs are more appropriate for persons with early dementia, and one-on-one interactions are geared toward persons with advanced dementia.^40,42^ Group programs, such as those incorporating music are most effective when they provide opportunities for one-on-one interactions for residents.^43^

Since residents with advanced dementia experience greater cognitive and physical limitations compared to those in the earlier stages of dementia, findings of the current study revealed the need to provide multiple activities to engage all of the senses of residents with the ultimate aim of promoting comfort. It is likely that greater effort by staff, volunteers, and families may be needed to meaningfully engage persons with advanced dementia who are at times considered a ‘hard to reach’ population. The benefits of multisensory stimulation are aligned with other studies as multisensory programs provide staff with access to a range of activities and opportunities to engage different senses.^15^ Bunn *et al.*^15^ found that through multisensory programs, staff become better able to deliver a range of activities which increases their knowledge and skills to respond to the changing needs and behaviors of residents. Maseda *et al.*^44^ similarly found that multisensory stimulation for older adults with advanced dementia led to feelings of enjoyment in activities, increased comfort, and reduced boredom.

### Implications for practice, policy, and research

With regard to practice implications, findings of the current study reveal the need for carefully planned staff training to improve a palliative approach to care through the Namaste Care program and a team effort in delivering the program. LTC staff require education and training in using various techniques to engage residents with advanced dementia to avoid a passive attempt at engagement.^24^ It is imperative that all staff, volunteers, and families understand the importance of meaningful engagement to increase the likelihood of the success of the Namaste Care program. A collaborative team approach with the ultimate goal of meaningfully engaging residents with advanced dementia needs to be implemented at all levels from management to bedside staff.

Policies that support the meaningful engagement of residents with advanced dementia by considering multiple environmental, social, and sensory factors should be made available in LTC. Namaste Care should be offered to a greater number of residents using a small group format for program delivery. There is a need for leadership to ensure adequate staff coverage so that they can focus on delivering Namaste Care. In LTC settings with limited resources, Namaste Care can require fewer resources if a group delivery format is used. Careful attention to equipment and space is required to determine where and how it is best to deliver Namaste Care. When persons with dementia transition from home to an LTC setting, there should be introduction guidelines available for families to ensure that residents will continue to participate in meaningful activities, even those near the end of life.^24^ This may include formally collecting information from families about the history, preferences, abilities, and interests of the person entering care.

Although data for this study were collected prior to the COVID-19 pandemic, findings reveal the increasing importance of meaningful engagement of residents with advanced dementia in LTC to offset negative effects of social isolation as a result of visitor restrictions and room confinement, such as anxiety, boredom, apathy, cognitive and physical decline, and depression.^24,45,46^ The research implication of the current study consists of measuring outcomes, such as quality of life, anxiety, and pain of residents, with advanced dementia to determine the effectiveness of the Namaste Care program. Future studies should compare whether an individual or group setting format for Namaste Care is most effective in meaningfully engaging residents with advanced dementia. Such studies need to ensure that program facilitators have knowledge of residents to engage them in a meaningfully way.

### Strengths and limitations

The strengths of the study were the large sample size and the inclusion of participants of diverse roles. Families, volunteers, administrators, and staff participated in this study to share their perceptions of Namaste Care and how it should be delivered. To the best of our knowledge, this is the first study to use the Comprehensive Process Model of Engagement^8^ to delineate the underlying influence of environmental, social, and sensory factors on LTC residents’ level of engagement in a Namaste Care program. There were, however, some limitations as only two LTC homes sites from one region in Canada were included and findings may therefore not be transferable to other contexts.

## Conclusion

This study revealed how environmental, social, and sensory factors influence the meaningful engagement of residents with advanced dementia in LTC. These factors should not be considered in isolation as they are often interrelated and impact one another. Going forward, LTC administrators and Namaste Care staff should consider providing individualized care to residents at the end stage of dementia and recognize that the needs and abilities of persons with dementia change over time and adapt the environment and stimulating activity accordingly. Namaste Care multisensory stimulation, in a small group format provided by staff who have knowledge of each residents’ unique needs and preferences, is important for the meaningful engagement of persons with advanced dementia.

## Supplemental Material

sj-docx-1-pcr-10.1177_26323524231165319 – Supplemental material for Exploring the factors influencing meaningful engagement of persons living with advanced dementia through the Namaste Care Program: a qualitative descriptive study Click here for additional data file. Supplemental material, sj-docx-1-pcr-10.1177_26323524231165319 for Exploring the factors influencing meaningful engagement of persons living with advanced dementia through the Namaste Care Program: a qualitative descriptive study by Marie-Lee Yous, Sheila A. Boamah, Paulette V. Hunter, Esther Coker, Thomas Hadjistavropoulos, Tamara Sussman and Sharon Kaasalainen in Palliative Care and Social Practice

## Declarations

**Ethics approval and consent to participate:** Ethical approval was granted from the Hamilton Integrated Research Ethics Board (#2865). All participants received a written introduction to the study and informed consent form written in lay language. Participants provided verbal or written consent.

**Consent for publication:** Not applicable.

**Availability of data and materials:** The data for this research consist of questionnaires, interview and focus group transcripts, and notes. Raw data cannot be publicly released due to the risk of compromising participant confidentiality.
