# Supplementary material for: Exploring the factors influencing meaningful engagement of persons living with advanced dementia through the Namaste Care Program: a qualitative descriptive study
Source: Palliat Care Soc Pract. 2023 Apr 1;17:26323524231165319. doi: 10.1177/26323524231165319 (PMC10071147; doi:10.1177/26323524231165319)
Supplement: sj-docx-1-pcr-10.1177_26323524231165319 – Supplemental material for Exploring the factors influencing meaningful engagement of persons living with advanced dementia through the Namaste Care Program: a qualitative descriptive study [file sj-docx-1-pcr-10.1177_26323524231165319.docx]

**Manuscript:** Exploring the Factors Influencing Meaningful Engagement of Persons Living with Advanced Dementia Through the Namaste Care Program: A Qualitative Descriptive Study

**Consolidated criteria for reporting qualitative studies (COREQ): 32-item checklist**

Developed from:

Tong A, Sainsbury P, Craig J. Consolidated criteria for reporting qualitative research (COREQ): A 32-item checklist for interviews and focus groups. *Int J Qual Health Care* 2007;19(6):349-357.

| **Topic** | **Item No.** | **Guide Questions/Descriptions** | **Reported on Page No.** |
| --- | --- | --- | --- |
| **Domain 1: Research team and reﬂexivity** | | | |
| *Personal characteristics* | | | |
| Interviewer/Facilitator | 1 | Which authors conducted the interview or focus group? | Research assistants - P. 5 |
| Credentials | 2 | What were the researchers’ credentials? (e.g. PhD, MD, MSc) | Title Page |
| Occupation | 3 | What were the researchers’ occupation at the time of the study? | Title Page |
| Gender | 4 | Were the researchers male or female? | Title Page |
| Experience and training | 5 | What experience or training did the researchers have? | P. 5 |
| *Relationship with participants* | | | |
| Relationship established | 6 | Was a relationship established prior to study commencement? | N/A |
| Participant knowledge of the interviewers | 7 | What did the participants know about the researchers? (e.g. personal goals, reasons for doing the research) | N/A |
| Interviewer characteristics | 8 | What characteristics were reported about the interviewers? (e.g. Bias, assumptions, reasons and interests in the research topic) | N/A |
| **Domain 2: Study design** | | | |
| *Theoretical framework* | | | |
| Methodological orientation and Theory | 9 | What methodological orientation was stated to underpin the study? (e.g. grounded theory, discourse analysis, ethnography, phenomenology, content analysis) | P. 4 |
| *Participant selection* | | | |
| Sampling | 10 | How were participants selected? (e.g. purposive, convenience, consecutive, snowball) | P. 4 |
| Method of approach | 11 | How were participants approached? (e.g. face-to-face, telephone, mail, email) | P. 4 |
| Sample size | 12 | How many participants were in the study? | P. 5 |
| Non-participation | 13 | How many people refused to participate or dropped out? Reasons? | N/A |
| *Setting* | | | |
| Setting of data collection | 14 | Where was the data collected? (e.g. home, clinic, workplace) | P. 4 |
| Presence of non-participants | 15 | Was anyone else present besides the participants and researchers? | N/A |
| Description of sample | 16 | What are the important characteristics of the sample? (e.g. demographic data, date) | P. 5 and Table 1 |
| *Data collection* | | | |
| Interview guide | 17 | Were questions, prompts, guides provided by the authors? Was it pilot tested? | P. 5 |
| Repeat interviews | 18 | Were repeat inter views carried out? If yes, how many? | N/A |
| Audio/visual recording | 19 | Did the research use audio or visual recording to collect the data? | P. 5 |
| Field notes | 20 | Were ﬁeld notes made during and/or after the interview or focus group? | P. 5 |
| Duration | 21 | What was the duration of the interviews or focus group? | P. 5 |
| Data saturation | 22 | Was data saturation discussed? | P. 5 |
| Transcripts returned | 23 | Were transcripts returned to participants for comment and/or correction? | N/A |
| **Domain 3: analysis and ﬁndings** | | | |
| *Data analysis* | | | |
| Number of data coders | 24 | How many data coders coded the data? | P. 5 |
| Description of the coding tree | 25 | Did authors provide a description of the coding tree? | N/A |
| Derivation of themes | 26 | Were themes identiﬁed in advance or derived from the data? | P. 5 |
| Software | 27 | What software, if applicable, was used to manage the data? | P. 5 |
| Participant checking | 28 | Did participants provide feedback on the ﬁndings? | N/A |
| *Reporting* | | | |
| Quotations presented | 29 | Were participant quotations presented to illustrate the themes/ﬁndings? Was each quotation identiﬁed? (e.g. participant number) | P. 5-10 |
| Data and ﬁndings consistent | 30 | Was there consistency between the data presented and the ﬁndings? | P. 5-10 |
| Clarity of major themes | 31 | Were major themes clearly presented in the ﬁndings? | P. 5-10 |
| Clarity of minor themes | 32 | Is there a description of diverse cases or discussion of minor themes? | P. 5-10 |
